# Efficient Underground Tunnel Place Recognition Algorithm Based on Farthest Point Subsampling and Dual-Attention Transformer

**DOI:** 10.3390/s23229261

**Published:** 2023-11-18

**Authors:** Xinghua Chai, Jianyong Yang, Xiangming Yan, Chengliang Di, Tao Ye

**Affiliations:** 1 54th Research Institute of China Electronics Technology Group Corporation, Shijiazhuang 050081, China; cxh88_88@163.com (X.C.); 13131128128@163.com (J.Y.); dichengliang@163.com (C.D.); 2 School of Mechanical Electronic and Information Engineering, China University of Mining and Technology, Beijing 100083, China; yanxmsir@163.com

**Keywords:** convolutional neural networks, underground tunnels, deep learning, place recognition

## Abstract

An autonomous place recognition system is essential for scenarios where GPS is useless, such as underground tunnels. However, it is difficult to use existing algorithms to fully utilize the small number of effective features in underground tunnel data, and recognition accuracy is difficult to guarantee. In order to solve this challenge, an efficient point cloud position recognition algorithm, named Dual-Attention Transformer Network (DAT-Net), is proposed in this paper. The algorithm firstly adopts the farthest point downsampling module to eliminate the invalid redundant points in the point cloud data and retain the basic shape of the point cloud, which reduces the size of the point cloud and, at the same time, reduces the influence of the invalid point cloud on the data analysis. After that, this paper proposes the dual-attention Transformer module to facilitate local information exchange by utilizing the multi-head self-attention mechanism. It extracts local descriptors and integrates highly discriminative global descriptors based on global context with the help of a feature fusion layer to obtain a more accurate and robust global feature representation. Experimental results show that the method proposed in this paper achieves an average $F_{1}$ score of 0.841 on the SubT-Tunnel dataset and outperforms many existing state-of-the-art algorithms in recognition accuracy and robustness tests.

## 1. Introduction

Place recognition is a fundamental capability for robot state estimation and is widely applied in robotic systems such as autonomous cars. In restricted environments like underground tunnels where GPS [1,2] is not available, place recognition can provide precise global positioning information for vehicles or robots, ensuring their stable operation in underground tunnels. Specifically, in stagewise pose estimation, place recognition adds loopback constraints to a vehicle’s repeated arrival intervals, reducing the cumulative error in globally consistent localization. Secondly, place recognition also helps initialize the positioning system for continuous posture tracking and is also responsible for acting as a backup positioning scheme in case of odometer drift. Therefore, the study of an efficient position identification method is of great significance in realizing global localization estimation [3] in underground tunnels. Existing 3D LiDAR-based position recognition methods have primarily been developed to address position recognition problems on urban roads. To the best of our knowledge, current 3D LiDAR-based position recognition methods have not been developed for underground tunnel environments and have not been tested on underground tunnel datasets.

Underground tunnels exhibit significant large-scale geometric repetition due to their unique scene configuration. Sensor data acquired in such environments inevitably lack effective geometric features that can be easily distinguished. Existing algorithms, which are mainly experimented with in usual environments such as school campuses and highways, generally rely on recognizable descriptive features in the data for place recognition. However, when faced with underground tunnel scenes with a large number of repeating 3D structures and similar textures, it is difficult to guarantee the original recognition accuracy. It is even more difficult to deal with the tricky situations that often occur in underground tunnels, such as obstacles blocking the sensors and changes in sensor viewpoints.

A few researchers have designed place recognition algorithms based on semantic information, focusing more on the influence of point cloud categories on the recognition effect. SG-PR [4], on the other hand, transforms point clouds into graph-structured data with the help of existing semantic segmentation methods. It describes the scene at the semantic level and focuses on the encoding relationships between semantic objects. However, the recognition accuracy of this method is heavily dependent on the performance of the segmentation algorithm, and the algorithm runs with high complexity. RINet [5] designs a rotationally invariant global descriptor and utilizes semantic information and geometric features to improve its discriminative ability. It has low computational complexity, but relies on highly accurate semantic labeling and spends extra time converting data formats. In addition, descriptor-based place recognition methods have also received extensive attention. M2DP [6] parses the point cloud from a multi-view perspective and obtains the density distribution of each point cloud based on the spatial density distribution characteristics of the points on the plane. The method is novel and robust, but it is difficult to guarantee the recognition accuracy due to the loss of some data features when projecting the point cloud data. SegMatch [7] segments the point cloud and drastically reduces the number of matches by clustering. It first encodes the features using a 3D convolutional neural network (CNN), after which the corresponding candidate values are identified through k-nearest neighbors (KNN), and finally, a geometric validation step is used to convert the candidate values into location-aware candidates. This approach combines the advantages of local and global descriptors, but the connection between objects is not fully considered, and the recognition accuracy needs to be improved. PointNetVLAD [8] innovatively adopts PointNet [9] as the backbone network to extract point cloud features, with targeted consideration of point cloud substitution invariance and affine invariance. PCAN [10] improves on the above method. It first extracts multi-scale local contextual information to generate point attention maps via the group sampling method of PointNet++ [11], and then uses NetVLAD [12] to aggregate the attention-weighted local features into a global descriptor. DAGC [13] again improves the above algorithm by combining the dynamic graph architecture and the dual-attention mechanism to aggregate local context information and then using the NetVLAD layer to aggregate the global descriptor. The above algorithm has been continuously improved and has gained some improvement in accuracy. However, the generalized PointNet-based point cloud analysis architecture makes it difficult to improve the recognition accuracy of complex underground scenes in a targeted manner. SeqLPD [14] and LPD-Net [15] first fuse the neighborhood features of each point in feature space and Cartesian space, and then use NetVLAD to generate global descriptors. However, some of the effective information is lost when 3D data are projected to 2D. SOE-Net [16] accomplishes the recognition task end-to-end. The network first extracts local descriptors point-by-point using the PointOE module, which is a combination of Pointnet and an Orientation-encoding Unit, and then aggregates differentiated global descriptors using the Self-Attention module and NetVLAD. However, the loss function threshold of this method needs to be set in advance.

In summary, some advanced algorithms rely on the accuracy of semantic segmentation algorithms, and it is difficult to independently and stably realize the autonomous recognition of underground tunnels. Meanwhile, the advanced place recognition methods based on descriptors make it difficult to ensure recognition accuracy due to the difficulty of fully utilizing the small number of effective features in the underground tunnel data. To address these issues, this paper introduces an efficient point cloud place recognition algorithm, DAT-Net. This method first employs a farthest point subsampling module to remove invalid redundant points from the point cloud data, reducing the point cloud scale while preserving its basic shape and geometric features to improve algorithm efficiency and convergence speed. Next, this paper proposes a dual-attention Transformer module, which utilizes dot-product attention and sinc attention to adaptively extract important features to enhance distortion sensitivity. Specifically, by using the simple and efficient parallelizable dot-product attention, attention is focused on the *K* key *V* value pairs related to the *Q* query vector, capturing local features in the data and adaptively enhancing local information in the point cloud. Additionally, the proposed sinc attention is used for local feature filtering, improving fine-grained quality prediction performance in underground tunnel scenes. This dual-attention strategy effectively enhances the extraction of local descriptors and integrates highly discriminative global descriptors based on the global context using the feature fusion layer, resulting in more accurate and robust global feature representation. Here are the contributions of this article:

1. The use of the farthest point subsampling module significantly reduces the point cloud size, decreasing the computational complexity of the model while preserving point cloud features.
2. A point cloud analysis module based on the dual-attention layer Transformer has been developed, enhancing the accuracy and robustness of place recognition.
3. Experiments in place recognition and robustness testing on an underground track dataset demonstrate that our approach performs exceptionally well, achieving an average dispersion of 0.841.

## 2. Model Design

### 2.1. Overall Network Design

**Figure 1:**
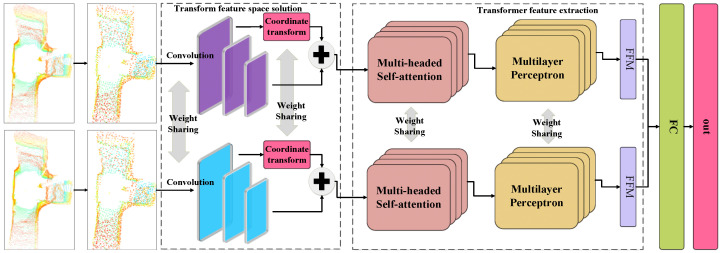
Place recognition method for efficient 3D point cloud (DAT-Net).

In order to improve the accuracy of the place recognition algorithm in underground tunnels, this paper proposes an efficient point cloud place recognition algorithm (DAT-Net), as shown in Figure 1. The algorithm first uses the farthest point downsampling module to eliminate the invalid redundant points in the point cloud data and retain the basic shape of the point cloud to reduce the size of the point cloud and at the same time reduce the impact of the invalid point cloud on the data parsing. After that, the transformed feature space solver module is used to dimensionally diffuse the point cloud and map the spatial flip suitable for feature extraction. Finally, the Transformer’s dual-attention layer is used to facilitate the exchange of multiple pieces of local information and integrate highly discriminative global descriptors according to the feature fusion module to obtain a more accurate and robust global feature representation.

### 2.2. Farthest Point Downsampling

Compared to images, a single frame of 3D LiDAR captures a significantly larger amount of data, and using this directly as input for a Transformer would lead to a substantial increase in model size. Refs [6,8,15] have used random downsampling methods or projected 3D data into 2D images to reduce the point cloud data volume. However, they lose too many point cloud features during downsampling, making it challenging to guarantee algorithm accuracy. Inspired by the research on PointNet [9], we believe that the outer points of an object hold more analytical value than the inner points because, in an ideal scenario, a sufficient number of outer points can adequately represent an object. Therefore, this paper adopts the farthest point downsampling algorithm, which reduces the scale of the point cloud while preserving environmental shape features as much as possible. The process is shown below:

1. Input: initial point cloud data {$p_{1},p_{2},\ldots ,p_{i}$}.
2. Select start point: randomly select a point as $p_{o}$ from the data or according to the density.
3. Initialize the subsampled point set: Begin by creating an empty subsampled point set *S*, and then add $P_{0}$ to the subsampled point set, resulting in *S* = {$P_{0}$}.
4. Loop until the termination condition is met:
  - For each point $p_{i}$ in the initial point cloud, compute the Euclidean distance $d_{i}$ from point $p_{o}$.
    $$d_{i}=\sqrt{{{[p}_{i\left(x\right)}-p_{o\left(x\right)}]}^{2}+{{[p}_{i\left(y\right)}-p_{o\left(y\right)}]}^{2}+{{[p}_{i\left(z\right)}-p_{o\left(z\right)}]}^{2}}$$
  - Sort the calculated Euclidean distance values and select the point furthest from point $p_{o}$ in the downsampled point set *S*.
  - Update the selected point to $p_{o}$.
5. Termination condition: the preset number of downsampled points *N* is reached.
6. Output: downsampled point cloud data *X*$\in R^{N\times 3}$.

**Figure 2:**
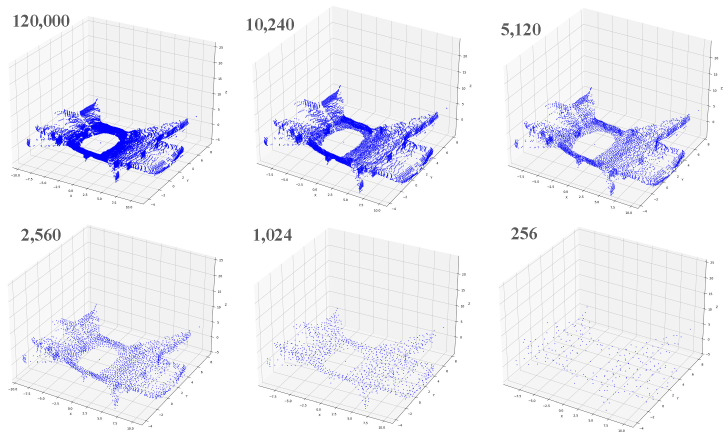
Effect of farthest point downsampling.

As shown in Figure 2, a frame of point cloud data from the SubT-Tunnel dataset typically contains around 120,000 points. Over-representation of the environment in some point-concentrated regions appears to be redundant. When the number of downsampling points *N* is set to 10,240 or 5120, it can be seen that the points in some areas are still very concentrated. After a series of attempts, it is found that when *N* is set to 1024, the downsampled point cloud can still express the shape of the original point cloud well, and will not be as sparse as the point cloud when *N* is set to 256. Therefore, this paper finally sets the number of downsampled points *N* to 1024. *N* = 1024 is the optimal value for this method. The specifications of the sensors and the environmental conditions during data collection influence the shape, density, and features of the original point cloud. The value of *N* for farthest point subsampling should be adjusted based on the specific environment and sensors.

### 2.3. Solution Module for Transformed Eigenspaces

Setting the step size of convolutional neural networks (CNNs) to 1 is capable of achieving translational isotropy in image analysis. However, in point clouds, 0 degrees and 359 degrees are adjacent, which means limiting the step size of CNNs to 1 does not fully realize rotational isotropy for point cloud analysis. Therefore, a convolutional neural network suitable for point cloud analysis is required to better advance the performance of place recognition. An intuitive solution is to use a recurrent convolutional neural network with a step size of 1. Its greatest property is rotational invariance, which is well-suited for point cloud analysis. First, the downsampled point cloud data $X\in R^{L\times 3}$ are subjected to three rotationally invariant convolution blocks, and then feature extraction is performed level by level to obtain different levels of features $F_{1}$, $F_{2}$, and $F_{3}$. The rotation invariant convolution can be expressed in the following form:

(1) $$\bar{V}(i)=[V\times K](i)=\sum_{c=0}^{N-1}\sum_{m=-M}^{M}V[(i-m)\mathrm{mod}N,c]\times K(M+m,c)$$

where i∈[0,N−1] and $K\in R^{(2M+1)\times N}$ denotes the convolution kernel.

**Figure 3:**
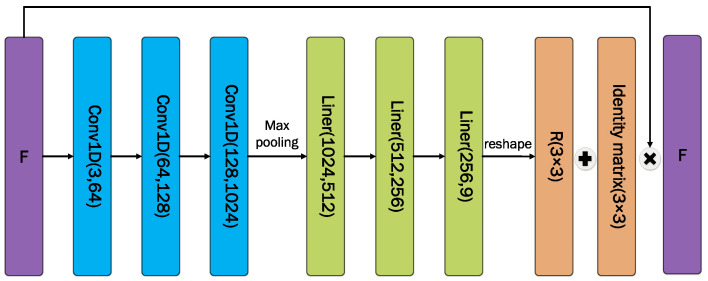
Coordinate transformation module.

As shown in Figure 3, the shallower feature $F_{1}$ is passed into the coordinate transformation module for feature dimension diffusion using 1D convolution. After that, the transformation matrix *R* is gathered and computed with the help of a linear connection layer. After flipping the feature $F_{1}$, it is fused with the deeper feature $F_{3}$ to enhance the adaptability of the algorithm to the scene perspective change.

### 2.4. Transformer

Transformer and self-attention mechanisms have made a number of contributions in the field of machine translation and NLP. This has inspired the development of self-attention models for images, the most influential of which is ViT [17]. Refs. [18,19] tried to analyze the whole point cloud with self-attention and found that its learning mechanism is very effective for point clouds. This paper introduces a dual-attention Transformer module, which utilizes both dot-product attention and sinc attention to adaptively extract important features, thereby enhancing sensitivity to distortions. Specifically, it focuses attention on *K* key–value pairs related to the *Q* query vector using the simple, efficient, and parallelizable dot-product attention. This helps capture local features in the data, adaptively reinforcing local information within the point cloud. Additionally, the proposed sinc attention is employed to perform local feature filtering and establish long-term dependencies, enhancing the algorithm’s fine-grained quality prediction performance in underground tunnel scenes. This dual-attention strategy effectively improves the extraction of local descriptors. Subsequently, with the help of the feature fusion layer, it integrates highly distinguishable global descriptors based on the global context to obtain a more accurate and robust global feature representation. More specifically, the feature fusion layer autonomously learns and assigns larger weight values to high-quality local descriptors, ensuring that the aggregated global descriptors can better describe the entire point cloud.

**Figure 4:**
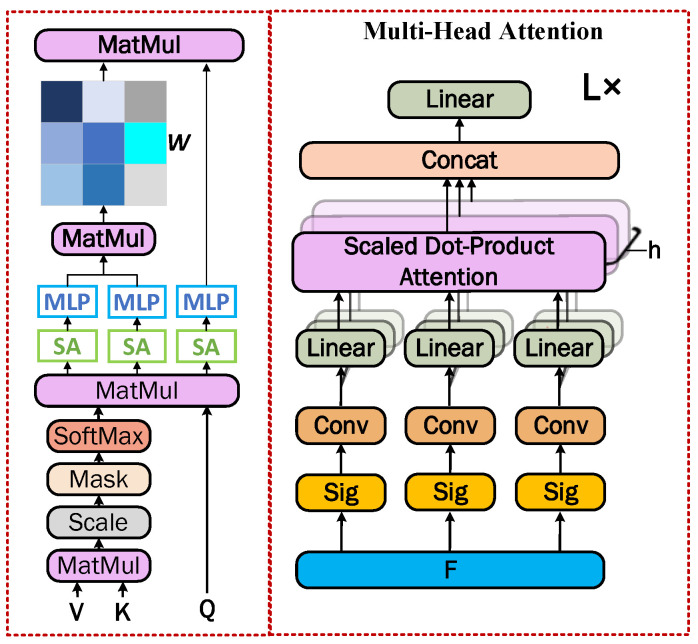
Multi-head self-attention layers.

The multi-head self-attention layer is shown in Figure 4. The feature *F* is first equated into local descriptors $Q\in R^{N\times D/h}$, $K\in R^{N\times D/h}$, and $V\in R^{N\times D/h}$ after a sigmoid layer, a rotationally invariant convolutional layer, and a linearly connected layer. Subsequently, the first local feature extraction is performed in the first attention layer. The computational process is represented by Equation (2) as follows:

(2) $$\begin{array}{c} Multihead(Q,K,V)=Contact(head_{1},\ldots ,head_{h})B^{O}\\ head_{i}=soft\max(\frac{QB_{i}^{Q}\cdot (KB_{i}^{K})}{\sqrt{D/h}})VB_{i}^{V} \end{array}$$

where *B* is the matrix of learnable parameters. $B_{i}^{Q}$, $B_{i}^{K}$, and $B_{i}^{V}\in R^{N\times D/h}$ and $B^{O}\in R^{D\times D}$.

The second attention layer is proposed to parse the point cloud features in more depth and is named as the Sine Attention Mechanism. It first substitutes features *Q*, *K*, and *V* into the Sinc function to obtain a smoother numerical representation. After that, the parameters are exchanged and shared through Multilayer Perceptron (*MLP*). Based on this, feature multiplication by *Q* and *K* is performed to calculate the assigned weights of local features. The formula for the sinusoidal attention mechanism is expressed as follows:

(3) $$\begin{array}{c} Multihead(Q,K,V)=Contact(head_{1},\ldots ,head_{h})\\ head_{i}=\frac{SA[MLP(Q_{i}K_{i}{}^{T})]}{\sum_{i=1}^{N}SA[MLP(Q_{i}K_{i}{}^{T})]}SA[MLP(V_{i})] \end{array}$$

The size of the features remains constant after multiple attention layers and the quality of the local descriptors can be improved by stacking the network layers L times. The final output is a splice of N D-dimensional local descriptors $F\in R^{N\times D}$. The h in the multi-head attention is set to 12 and the number of loops L is also set to 12.

### 2.5. Feature Fusion Module

Inspired by SimGNN [20] and SG-PR [4], this paper assigns weights to local descriptors with the help of the attention mechanism. It learns autonomously and assigns larger weight values to high-quality local descriptors so that the aggregated global descriptors can better describe the whole point cloud.

**Figure 5:**
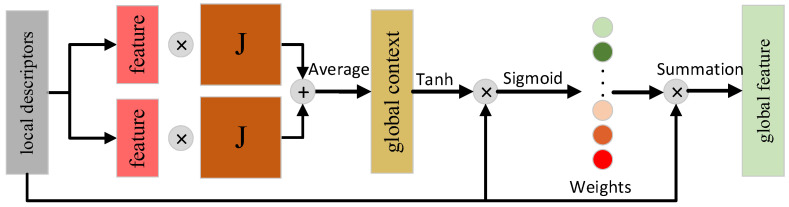
Feature fusion module.

As shown in Figure 5, the learnable parameter matrix $J\in R^{D\times D}$ is set. First, *J* is multiplied with the average of all local descriptors to construct a global context to provide global structure and feature information. After that, a sigmoid function is used to ensure that the weights are in [0, 1] to assign a weight value to each local descriptor. Finally, the final global descriptor $e\in R^{D}$ is obtained through summation, which is expressed by the following:

(4) $$\begin{array}{c} e=\sum_{i=1}^{N}a_{i}F_{i}\\ a_{i}=sigmoid\left(F_{i}\cdot \tanh\left(contact{\left(\frac{1}{N}\sum_{m=1}^{N}U_{m}^{1}J_{1},\frac{1}{N}\sum_{m=1}^{N}U_{m}^{2}J_{2}\right)}^{T}\right)\right) \end{array}$$

### 2.6. Loss Function

In this paper, two linear connected layers are used to reduce the dimensionality of the matching vectors, and a sigmoid function is used to obtain the similarity $p(y_{i})\in [0,1]$ of the scenes. The loss function used for training is the binary cross-entropy loss function with the following equation:

(5) $$L\mathrm{oss}=-\frac{1}{N}\sum_{i=1}^{N}y_{i}\cdot \log[p(y_{i})]+(1-y_{i})\cdot \log[1-p(y_{i})]$$

where $y_{i}$ is the binary label 0 or 1, and $p(y_{i})$ is the probability that the output belongs to label $y_{i}$.

## 3. Test Results and Performance Analysis

In order to evaluate the effectiveness of the method proposed in this paper, the experiments are conducted using the SubT-Tunnel [21] underground tunnel dataset, which consists of three different tunnel scenarios, S1, S2, and E1. The server for training uses an Intel^®^ CoreTM i7-6950X CPU processor with four NVIDIA GeForce GTX 1080Ti, each with 11G of memory. The experiments are implemented on a Pytorch [22] deep learning framework based on the Ubuntu 18.04 operating system with GPU (GTX1080Ti) for training and testing. In this paper, the model parameters are optimized using the Adam [23] optimizer, where the initial learning rate is set to 0.0001, learning decreases with the number of iterations, weight decay is 0.0005, batch size is 1024, and the maximum number of iterations is 500 rounds.

### 3.1. Experimental Setup

In the experiments, point cloud pairs with timestamps differing by more than 30 s and Euclidean distances less than 3 m will be defined as positive samples, and point cloud pairs with Euclidean distances more than 20 m will be defined as negative samples. During training, sample pairs with timestamps less than 30 s are also included in the training set to increase the interference term. During the evaluation process, positive and negative samples with a time difference of less than 30 s are eliminated. This means that the performance of the algorithm is not evaluated based on simple pairs of positive samples (neighboring scenarios), allowing for a more accurate assessment of its capabilities in real-world environments. In this paper, DAT-Net is compared with state-of-the-art algorithms such as M2DP [6], LPD-NET [15], DISCO [24], PointNetVLAD [8], SeqOT [25], and RINET [5]. For a fair comparison, the $F_{1}$ score [26] value is used uniformly to measure the algorithm performance. $F_{1}$ is defined as follows, where *P* denotes precision and *R* denotes recall.

(6) $$F_{1}=\frac{2\times P\times R}{P+R}$$

### 3.2. Analysis of Experimental Results

**Figure 6:**
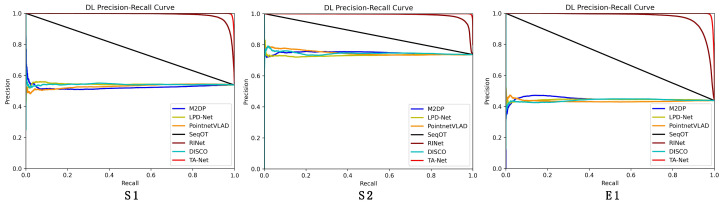
Accuracy–recall for place recognition.

Qualitative Analysis: Figure 6 shows the recall–precision plot, and the P-R curve of DAT-Net shows that it has higher recall and precision compared to the other algorithms in each scene, which indicates that the method proposed in this paper is able to maintain better recognition in different underground tunnel scenes. In addition, it can be seen that unlike the S1 and S2 scenarios, the performance of each algorithm decreases somewhat in the mega-scene E1. However, the proposed algorithm DAT-Net maintains a stable performance in the E1 scenario compared to other state-of-the-art algorithms, which indicates that the accuracy and stability of DAT-Net are excellent.

Quantitative Analysis: According to the results in Table 1, DAT-Net achieved excellent experimental results on the underground tunnel dataset, i.e., an average maximum score of 0.841. In this experiment, DAT-Net scored on average 0.134 points higher than the second-place RINet in the S1, S2, and E1 scenarios. The experimental results of M2DP and LPD-Net were unsatisfactory, mainly because they converted the data to top view, resulting in the loss of some features of the underground tunnel data. SeqOT also lost some valid information in the data conversion. PointNetVLAD only considers the features of a single point and ignores their connection with neighboring features. Underground tunnel scenes have more sparse effective features compared to normal scenes, so it is difficult to use the above algorithms to show higher accuracy in underground tunnels due to the lack of effective utilization of features. It is worth noting that the E1 series used in the experiments were recorded in an ultra-large-scale complex underground tunnel environment, and all the algorithms decreased in accuracy for this series. DAT-Net still obtained a high score as high as 0.797 in the E1 scene, indicating that the method proposed in this paper still has excellent recognition capability and stability in large underground tunnel scenes.

### 3.3. Robustness Testing

Obstacle occlusion and sensor view angle changes are issues that need to be addressed in the daily tasks of autonomous driving.

**Figure 7:**
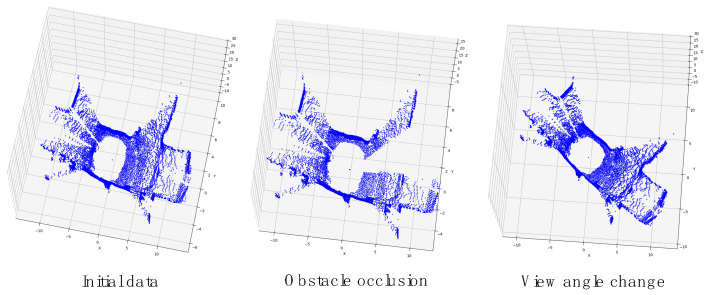
Schematic diagram of obstacle occlusion and view angle change.

As shown in Figure 7, obstacle occlusion directly leads to incomplete data information, and the change of view angle is not visible from the human perspective, but it is a more drastic change to the original data for the electronic processor. In order to verify the impact of the above problems on the place recognition algorithm and to further test the stability and robustness of the algorithm, this paper simulates the underground tunnel scene with obstacle occlusion and view angle change to compare the experiment with other state-of-the-art algorithms.

Occlusion test: Sensor occlusion is a common phenomenon in automobile and robot operation that can easily lead to incomplete data acquired by sensors. For place recognition, using data with missing important information features can significantly affect the algorithm performance. In order to evaluate the processing ability of different methods for defective data, in this paper, points in a region are randomly removed from the point cloud. The results shown in Table 2 indicate that the method proposed in this paper significantly outperforms the other algorithms in this experiment, exceeding the second-place algorithm, RINet, by 0.340 on average. It exceeds by 0.234 in the S1 scenario, 0.244 in the S2 scenario, and 0.244 in the E1 scenario. This shows that the missing information has a great impact on RINet, which prevents it from maintaining its performance in normal situations. The DAT-Net method proposed in this paper reduces the amount of point cloud data in the data preprocessing stage and maintains the data features as much as possible, which effectively mitigates the performance degradation of the algorithm due to the missing information. This demonstrates that the method proposed in this paper also exhibits strong robustness in environments with obstacle occlusion.

Rotation test: Sensor viewpoint changes are also a common phenomenon in autonomous driving. For the human eye, it is not difficult to recognize a scene that is at a different view angle when passing by the same place. However, for position recognition, the change in view angle brings a completely different form of data. In order to evaluate the ability of the different methods to handle changes in sensor view angle, this paper randomly rotates each point in the point cloud. The results shown in Table 3 indicate that DAT-Net still significantly outperforms the other algorithms in the experiments, exceeding the second-place algorithm RINet by 0.246 on average. It exceeds by 0.256 in the S1 scene, 0.241 in the S2 scene, and 0.246 in the E1 scene. RINet produces a more severe accuracy degradation under the influence of perspective changes. The reason for this is that the missing information only affects part of the data, while the change in view angle changes all the data. The proposed method, DAT-Net, uses the transformed feature space solver module to autonomously learn favorable transform matrices to enhance the parsing effect on the data, which effectively mitigates the algorithm’s performance degradation caused by changes in the sensor’s view angle.

The obstacle occlusion and viewpoint change tests demonstrate the stability and robustness of the method proposed in this paper for underground tunnel scenarios.

### 3.4. Algorithm Efficiency

As shown in Table 4, our method has 3.18 M parameters, 1.12 GFLOPs, and a recognition time of 36.0 ms for a single frame of data. Taking the TX2 embedded device used in practical scenarios as an example, with its hardware performance reaching 1.3 TFLOPS, it is more than sufficient to meet the operational requirements of our method. Additionally, our algorithm also fulfills the fundamental real-time processing needs.

## 4. Conclusions

Using the existing algorithms, it is difficult to fully utilize the small number of effective features in the underground tunnel data, and the recognition accuracy is difficult to guarantee for other problems. This paper proposes an efficient underground tunnel location identification algorithm (DAT-Net) that combines point cloud preprocessing and a dual-attention Transformer. The algorithm firstly adopts the farthest point downsampling module to eliminate the invalid redundant points in the point cloud data and retain the basic shape of the point cloud so as to reduce the size of the point cloud and at the same time reduce the influence of the invalid point cloud on the data parsing. After that, the dual-attention Transformer module is proposed to utilize the multi-head self-attention mechanism to promote local information exchange and extract local descriptors. With the help of the feature fusion layer, the highly recognizable global descriptors are integrated according to the global context, obtaining a more accurate and robust global feature representation. The experimental results show that the method proposed in this paper achieves an average $F_{1}$ score of 0.841 on the NVIDIA GTX 1080Ti experimental platform for the underground tunnel dataset, and it performs well in the recognition accuracy and robustness tests compared to many existing state-of-the-art algorithms. Therefore, it is able to meet the demand for high accuracy and robust position recognition for complex underground tunnel scenes.

## Figures and Tables

**Table 1:** Average $F_{1}$ scores of each advanced algorithm on SubT-Tunnel dataset.

Methods	S1	S2	E1	Mean
M2DP	0.456	0.550	0.428	0.478
LPD-Net	0.472	0.540	0.426	0.479
SeqOT	0.350	0.424	0.305	0.360
DISCO	0.472	0.547	0.424	0.481
PointNetVLAD	0.466	0.545	0.415	0.475
RINet	0.716	0.706	0.700	0.707
DAT-Net (Our)	0.837	0.888	0.797	0.841

**Table 2:** Average $F_{1}$ scores of each advanced algorithm for the occlusion test.

Methods	S1	S2	E1	Mean
M2DP	0.489	0.553	0.410	0.484
LPD-Net	0.481	0.543	0.422	0.482
SeqOT	0.350	0.424	0.305	0.360
DISCO	0.475	0.501	0.421	0.466
PointNetVLAD	0.475	0.553	0.417	0.476
RINet	0.567	0.610	0.520	0.566
DAT-Net (Our)	0.801	0.854	0.764	0.806

**Table 3:** Average $F_{1}$ scores for each advanced algorithm for rotational testing.

Methods	S1	S2	E1	Mean
M2DP	0.456	0.549	0.428	0.478
LPD-Net	0.461	0.544	0.418	0.474
SeqOT	0.350	0.424	0.305	0.360
DISCO	0.476	0.540	0.421	0.479
PointNetVLAD	0.464	0.541	0.417	0.474
RINet	0.501	0.585	0.476	0.521
DAT-Net (Our)	0.757	0.823	0.722	0.767

**Table 4:** Efficiency demonstration of various advanced algorithms.

Methods	Parameters (M)	FLOPs (G)	Runtime (ms)
LPD-Net	2.49	5.51	42.3
SeqOT	1.99	0.67	27.0
DISCO	7.79	1.33	38.3
PointNetVLAD	2.81	0.90	28.4
RINet	1.82	0.058	10.7
DAT-Net (Our)	3.18	0.112	36.0

## Data Availability

The SubT-Tunnel dataset can be obtained from the following website: https://ieeexplore.ieee.org/abstract/document/9197156, accessed on 15 September 2020.
